# Supplementary material for: Impact of Ectropis grisescens Warren (Lepidoptera: Geometridae) Infestation on the Tea Plant Rhizosphere Microbiome and Its Potential for Enhanced Biocontrol and Plant Health Management
Source: Insects. 2025 Apr 14;16(4):412. doi: 10.3390/insects16040412 (PMC12027819; doi:10.3390/insects16040412)
Supplement: Supplementary file 1 [file insects-16-00412-s001.zip › insects-3517371-supplementary.pdf]

Supplementary Figure S1:

The results of the tablet induced growth experiment：

Different numbers represent the treatment of tomato seeds with different bacterial solutions, and sterile water is used as a reference to measure the growth indicators of tomato seeds

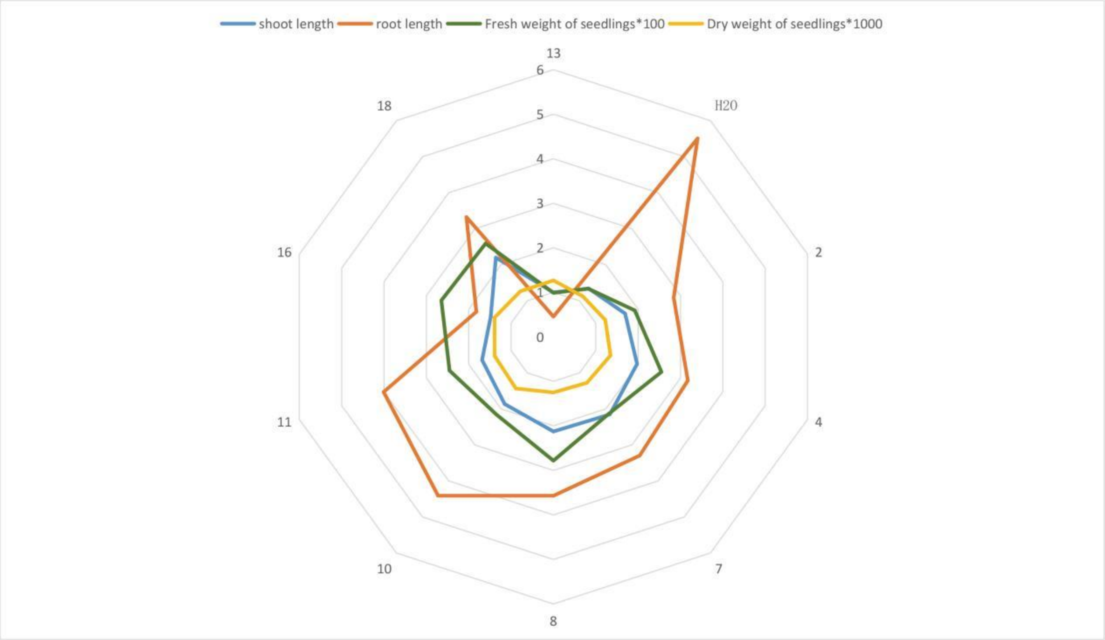

Supplementary Table S1:
The number of enriched-KOs in infested samples in rhizosphere at different level of KEGG pathway.

Pathway ID	Level1	Level2	Level3	The number of enriched--KOs
ko00260	Metabolism	Amino acid metabolism	Glycine, serine and threonine metabolism	4
ko00270	Metabolism	Amino acid metabolism	Cysteine and methionine metabolism	1
ko00300	Metabolism	Amino acid metabolism	Lysine biosynthesis	1
ko00330	Metabolism	Amino acid metabolism	Arginine and proline metabolism	2
ko00350	Metabolism	Amino acid metabolism	Tyrosine metabolism	1
ko00360	Metabolism	Amino acid metabolism	Phenylalanine metabolism	2
ko00380	Metabolism	Amino acid metabolism	Tryptophan metabolism	2
ko00400	Metabolism	Amino acid metabolism	Phenylalanine, tyrosine and tryptophan biosynthesis	2
ko00920	Metabolism	Amino acid metabolism	Sulfur metabolism	1
ko00030	Metabolism	Amino acid metabolism	Pentose phosphate way	3
ko00362	Metabolism	Amino acid metabolism	Benzoate degradation	2
ko00310	Metabolism	Amino acid metabolism	Lysine degradation	2
ko00333	Metabolism	Biosynthesis of other secondary metabolites	Prodigiosin biosynthesis	1
ko00030	Metabolism	Carbohydrate metabolism	Pentose phosphate way	
ko00362	Metabolism	Carbohydrate metabolism	Benzoate degradation	2
ko00010	Metabolism	Carbohydrate metabolism	Glycolysis / Gluconeogenesis	3
ko00040	Metabolism	Carbohydrate metabolism	Pentose and glucuronate interconversions	1
ko00051	Metabolism	Carbohydrate metabolism	Fructose and mannose metabolism	3
ko00052	Metabolism	Carbohydrate metabolism	Galactose metabolism	1
ko00062	Metabolism	Carbohydrate metabolism	Fatty acid elongation	1
ko00071	Metabolism	Carbohydrate metabolism	Fatty acid degradation	1
ko00280	Metabolism	Carbohydrate metabolism	Valine, leucine and isoleucine degradation	1

ko00290	Metabolism	Carbohydrate metabolism	Valine, leucine and isoleucine biosynthesis	1
ko00500	Metabolism	Carbohydrate metabolism	Starch and sucrose metabolism	1
ko00520	Metabolism	Carbohydrate metabolism	Amino sugar and nucleotide sugar metabolism	3
ko00620	Metabolism	Carbohydrate metabolism	Pyruvate metabolism	3
ko00640	Metabolism	Carbohydrate metabolism	Propanoate metabolism	1
ko00650	Metabolism	Carbohydrate metabolism	Butanoate metabolism	2
ko00930	Metabolism	Carbohydrate metabolism	Caprolactam degradation	1
ko00310	Metabolism	Carbohydrate metabolism	Lysine degradation	2
ko00030	Metabolism	Energy metabolism	Pentose phosphate way	
ko00362	Metabolism	Energy metabolism	Benzoate degradation	2
ko00190	Metabolism	Energy metabolism	Oxidative phosphorylation	1
ko00680	Metabolism	Energy metabolism	Methane metabolism	4
ko00910	Metabolism	Energy metabolism	Nitrogen metabolism	5
ko00623	Metabolism	Energy metabolism	Toluene degradation	2
ko00552	Metabolism	Glycan biosynthesis and metabolism	Teichoic acid biosynthesis	1
ko00571	Metabolism	Glycan biosynthesis and metabolism	Lipoarabinomannan (LAM) biosynthesis	1
ko00121	Metabolism	Lipid metabolism	Secondary bile acid biosynthesis	1
ko00564	Metabolism	Lipid metabolism	Glycerophospholipid metabolism	1
ko00310	Metabolism	Lipid metabolism	Lysine degradation	2
ko00730	Metabolism	Metabolism of cofactors and vitamins	Thiamine metabolism	2
ko00750	Metabolism	Metabolism of cofactors and vitamins	Vitamin B6 metabolism	2
ko00790	Metabolism	Metabolism of cofactors and vitamins	Folate biosynthesis	1
ko00860	Metabolism	Metabolism of cofactors and vitamins	Porphyrin metabolism	2
ko00410	Metabolism	Metabolism of cofactors and vitamins	beta-Alanine metabolism	1
ko00440	Metabolism	Metabolism of other amino acids	Phosphonate and phosphinate metabolism	1
ko00410	Metabolism	Metabolism of other amino acids	beta-Alanine metabolism	1

ko00770	Metabolism	Metabolism of terpenoids and polyketides	Pantothenate and CoA biosynthesis	1
ko00906	Metabolism	Metabolism of terpenoids and polyketides	Carotenoid biosynthesis	3
ko00907	Metabolism	Metabolism of terpenoids and polyketides	Pinene, camphor and geraniol degradation	2
ko00908	Metabolism	Metabolism of terpenoids and polyketides	Zeatin biosynthesis	1
ko00983	Metabolism	Metabolism of terpenoids and polyketides	Drug metabolism - other enzymes	1
ko00310	Metabolism	Metabolism of terpenoids and polyketides	Lysine degradation	2
ko00410	Metabolism	Nucleotide metabolism	beta-Alanine metabolism	1
ko00230	Metabolism	Nucleotide metabolism	Purine metabolism	4
ko00240	Metabolism	Nucleotide metabolism	Pyrimidine metabolism	5
ko00622	Metabolism	Nucleotide metabolism	Xylene degradation	1
ko00643	Metabolism	Nucleotide metabolism	Styrene degradation	1
ko00623	Metabolism	Xenobiotics biodegradation and metabolism	Toluene degradation	2
ko00410	Metabolism	Xenobiotics biodegradation and metabolism	beta-Alanine metabolism	1
ko00361	Metabolism	Xenobiotics biodegradation and metabolism	Chlorocyclohexane and chlorobenzene degradation	2
ko00627	Metabolism	Xenobiotics biodegradation and metabolism	Aminobenzoate degradation	2
ko00310	Metabolism	Xenobiotics biodegradation and metabolism	Lysine degradation	2

Supplementary Table S2：accession number GSA

MN661273.1	Burkholderia cepacia strain ABC4
MK418965.1	Burkholderia cepacia strain B-6
KY810685.1	Burkholderia cenocepacia strain MNR-239
OQ940509.1	Burkholderia sp. strain HAU-B3
LN889999.1	Burkholderia ambifaria strain L3
MN759657.1	Burkholderia sp. strain BW-1
MN044776.1	Burkholderia sp. strain S1
KU042966.1	Burkholderia sp. MQ-14W
MG452786.1	Burkholderia sp. strain JXDG201608-14
EF394149.1	Bradyrhizobium liaoningense strain CCBAU 43298
